# Predictive Utility of Near-Infrared Spectroscopy for the Outcomes of Hypoxic-Ischemic Encephalopathy: A Systematic Review and Meta-Analysis

**DOI:** 10.7759/cureus.51162

**Published:** 2023-12-27

**Authors:** Khaled M El-Atawi, Mohammed F Osman, Moustafa Hassan, Zohra A Siwji, Ahmed A Hassan, Maysam Y Abed, Yasser Elsayed

**Affiliations:** 1 Pediatrics/Neonatal Intensive Care Unit, Latifa Women and Children Hospital, Dubai, ARE; 2 Neonatology, Tawam Hospital, Al Ain, ARE; 3 Neonatology, Dubai Hospital, Dubai, ARE; 4 Pediatric Cardiology, Al Jalila Children's Speciality Hospital, Dubai, ARE; 5 Pediatric Cardiology, Ibn Albitar Tertiary Center for Cardiology, Baghdad, IRQ; 6 Pediatrics and Neonatology, Health Sciences Centre-Winnipeg, Max Rady College of Medicine, University of Manitoba, Manitoba, CAN

**Keywords:** meta-analysis, systematic review, perinatal outcomes, hypoxic-ischemic encephalopathy, near-infrared spectroscopy

## Abstract

This systematic review and meta-analysis aimed to assess the utility of near-infrared spectroscopy (NIRS) in predicting the perinatal outcomes of neonates with hypoxic-ischemic encephalopathy (HIE). We conducted a literature search on Medline via PubMed, Web of Science, Scopus, and CENTRAL Library. We included studies that utilized early NIRS monitoring to study the accuracy of NIRS in predicting the perinatal outcomes of neonates with hypoxic-ischemic encephalopathy. Nine studies that met our eligibility criteria were included. These studies were published between 2012 and 2023. In this meta-analysis, no significant differences in regional cerebral oxygen saturation (cSpO2) were found between normal and abnormal groups at 12 hours (MD = 0.21, 95% CI: -6.39 to 6.82, P = 0.95) and 24 hours (MD = -1.96, 95% CI: -6.95 to 3.03, P = 0.44). However, at 48 hours, cSpO2 was significantly lower in the normal group (MD = -4.9, 95% CI: -5.91 to -3.89, P < 0.00001). At 72 hours, our analysis revealed a significant difference with lower cSpO2 in the normal group (MD = -3.0, 95% CI: -5.5 to -0.5, P = 0.02). Regarding cerebral fractional tissue oxygen extraction (FTOE), no significant differences were observed at 12 hours (MD = 0.03, 95% CI: -0.02 to 0.09, P = 0.24). After 24 hours, the normal group exhibited lower FTOE (MD = -0.03, 95% CI: -0.04 to -0.01, P < 0.001), while after 48 hours, the normal group had higher FTOE (MD = 0.07, 95% CI: 0.04 to 0.10, P < 0.0001). Early cerebral NIRS monitoring is beneficial in predicting the outcomes of HIE in term neonates. Our analysis showed that several NIRS parameters, such as regional cSpO2 and cerebral FTOE, are significantly associated with adverse outcomes in the first 72 hours of birth.

## Introduction and background

Hypoxic-ischemic encephalopathy (HIE) is a leading cause of neonatal mortality and long-term developmental abnormalities worldwide. According to previous epidemiological studies, HIE affects 1-8 per 1000 live births, increasing to 26 per 1000 in low socioeconomic countries [1]. The condition develops secondary to inadequate cerebral blood flow due to a hypoxic event, leading to impaired fetal cardiac output and structural damage to the brain. Several risk factors were incorporated in the development of the hypoxic event, such as placental abruption and uterine rupture [2]. The extent of brain injury depends on the onset and severity of the hypoxic event, leading to either a circulatory shunt and limited brain injury or a sudden drop in cerebral blood flow and extensive damage [3]. Besides, the extent of the injury and deployed interventions largely determine the recovery phase and the risk of neurological outcomes [4]. Despite the widespread use of hypothermia and recent advances in HIE therapeutics, several reports still suggest suboptimal outcomes of HIE in many term infants. Neonates with moderate-to-severe HIE are at significant risk of seizures, cerebral palsy, other adverse neuro disabilities, and multiorgan failure [5]. Moreover, HIE accounts for nearly 15% of the mortality among children under five years [6]. Thus, timely diagnosis, outcome prediction, and response identification are crucial to optimize the management of HIE.

Several biomarkers have been recently investigated to predict HIE outcomes in term neonates. Combining electroencephalography findings and clinical examination is the cornerstone for outcome prediction in HIE [7]. However, previous reports demonstrated that the utility of amplitude-integrated EEG (aEEG) in the early prediction of HIE outcomes was limited by hypothermia treatment [8]. Furthermore, the clinical usefulness of serum biomarkers for HIE has not been well established yet [2]. Cerebral near-infrared spectroscopy (NIRS) is a noninvasive neuromonitoring modality that accurately reflects a disturbance in blood flow, oxygenation, and metabolism from birth. Cerebral NIRS is an easy-to-use, quick, and inexpensive modality that utilizes NIR light across the transparency of biological tissue; neonates are ideal candidates for NIRS monitoring due to their thin skin and skull, allowing for deeper penetration into brain structures [9,10]. Recent evidence has highlighted that NIRS monitoring can play a beneficial role in assessing cerebral perfusion and oxygenation and evaluating neonatal cerebral autoregulation in the setting of HIE. It was also found that NIRS monitoring can significantly predict neurodevelopmental outcomes [11]. However, the current evidence is inconclusive regarding the utility of early NIRS monitoring in predicting outcomes of HIE [12,13]. Thus, we conducted the present systematic review and meta-analysis to assess the usefulness of NIRS in predicting the perinatal outcomes of neonates with HIE.

## Review

Materials and methods

We conducted the present study according to the Preferred Reporting Items for Systematic Reviews and Meta-Analyses (PRISMA) guideline [14] and the Cochrane Handbook for Systematic Reviews of Interventional [15].

Literature Search

We conducted a literature search from inception to October 2023 in MEDLINE via PubMed, Web of Science, Scopus, and CENTRAL Library. We searched both the title and abstract using the following keywords: ((hypoxic-ischemic encephalopathy [MeSH Terms]) OR (hypoxic ischemic encephalopathies [MeSH Terms]) OR hypoxic-ischemic encephalopathy OR HIE) AND (near-infrared spectroscopy [MeSH Terms] OR near infrared spectroscopies [MeSH Terms] OR NIRS) AND (mortality, perinatal [MeSH Terms] OR outcome). We retrieved the relevant citations using the Endnote X9 software package (Thomson Reuters, USA).

Eligibility Criteria

Retrieved records were screened using the following criteria: (I) studies that included late preterm/term neonates (gestational age ≥36 weeks) with HIE, which was defined according to the Thompson score (18), Sarnat score (19), or electroencephalogram; (II) studies that utilized early NIRS monitoring (within six hours of age); (III) studies that compared NIRS parameters between neonates with adverse and non-adverse perinatal outcomes; and (IV) studies that were prospective or retrospective cohort, case-control, cross-sectional studies, or case series. We excluded experimental studies, non-original publications, theses, conference proceedings, and non-English language articles and studies. The screening was performed in a two-step process, i.e., title/abstract screening and full-text screening. Each step was done independently by two reviewers according to the pre-specified criteria.

Data Extraction and Quality Assessment

We extracted the characteristics of each study as follows: first author, year of publication, study design, population size, HIE characteristics, use of magnetic resonance imaging (2), NIRS parameters, perinatal outcomes, and main findings of the studies. The NIRS measures included tissue oxygen extraction (3), cerebral oxygen saturation (cSpO2), cerebral blood volume (CBV), and fractional oxygen extraction (FTOE). Consequently, the perinatal outcomes included MRI-based injury and neurodevelopmental outcomes. The risk of bias of included studies was assessed using the Quality Assessment of Diagnostic Accuracy Studies (QUADAS-2) tool.

Statistical Analysis

We conducted pairwise comparisons using Review Manager (RevMan, Cochran Collaboration) version 5.4 to pool studies. We used mean difference (MD) or standardized mean difference (SMD) and 95% confidence interval (CI) to assess the difference in NIRS parameters between neonates with and without adverse perinatal outcomes. We assessed statistical heterogeneity using the chi-square test, and its extent was measured using the I-square test. We used the random-effects model if there was heterogeneity between studies (I^2^ < 50%); otherwise, the fixed-effects model was used (I^2^ < 50%).

Results

Search Strategy Results

**Figure 1:**
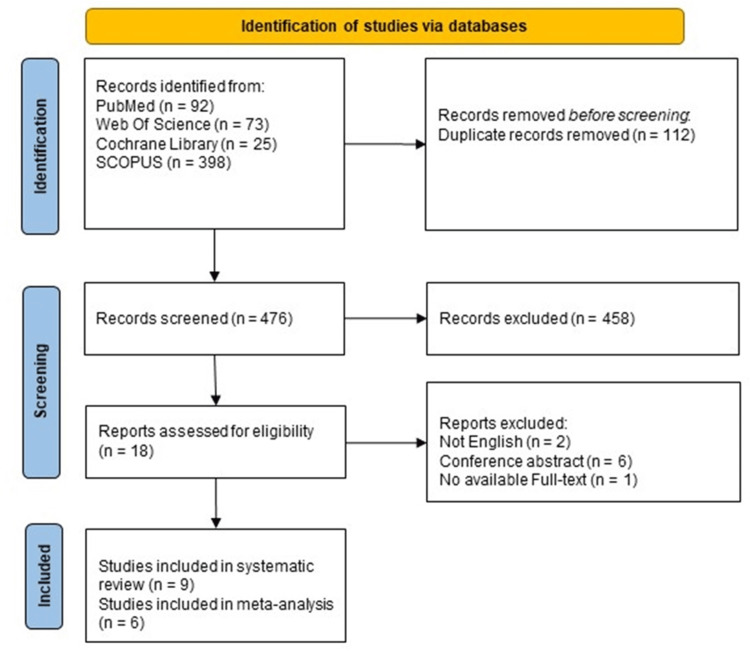
PRISMA flow diagram. PRISMA: Preferred Reporting Items for Systematic Reviews and Meta-Analyses.

Initially, 588 citations were retrieved and imported into the Endnote X8 program. Of them, 476 records were screened for eligibility, of which 18 were retained for full-text screening. Overall, nine observational studies were included in the present review, and six studies were included in the meta-analysis (Figure 1).

Study characteristics and quality assessment*: *Nine studies published between 2012 and 2021 were included in the present study. Seven studies were prospective, and two were retrospective chart reviews [16,17]. The studies mainly included patients with moderate-to-severe HIE, with a sample size ranging from 10 to 666 patients. All studies, except Ancora et al. [18], utilized brain MRI for injury assessment within the first two weeks. Additionally, five studies used scoring systems for the assessment of developmental outcomes. The studies show variable conclusions regarding the predictive utility of NIRS for the outcomes of neonates with HIE (Table 1).

**Table 1:** Summary Characteristics of the Included Studies HIE: hypoxic-ischemic encephalopathy, MRI: magnetic resonance imaging, cSPO2: cerebral saturation of peripheral oxygen, rSO2: regional oxygen saturation, cFTOE: cerebral fractional tissue oxygen extraction, TOI: tissue oxygenation index, ΔHbO2: change in oxyhemoglobin, ΔHb: change in deoxyhemoglobin, FTOE: fractional tissue oxygen extraction.

Author	Year	Design	Population	N	Outcome Measures	Main Findings
Nakamura et al. [16]	2015	Retrospective study	Neonates with HIE	11	MRI at weeks 1–2; Psychological development 1–1.5 years	Cerebral blood volume and cSPO2 significantly predict adverse outcomes at 24 h
Ze et al. [17]	2021	Retrospective study	Neonates with HIE	666	Mean rSO2 and cFTOE on the first date	The rSO2 and cFTOE levels in normal neonates from high-altitude areas were lower than in neonates from low-altitude areas
Ancora et al. [18]	2013	Prospective study	Neonates with moderate to severe HIE	12	Griffiths, at 1.5 years	Tissue Oxygenation Index (TOI) had no predictive value in predicting outcomes in HIE
Liu et al. [19]	2020	Prospective study	Neonates with HIE	57	Oxyhemoglobin (ΔHbO2) and deoxyhemoglobin (ΔHb)	Neonates showed the early ability to differentiate among emotional prosodies, responding most sensitively to positive emotions
Niezen et al. [20]	2018	Prospective study	Asphyxiated newborns treated with hypothermia	39	MRI at days 4–13; Beyleys at 2 years	cSPO2 had no predictive value in predicting outcomes in HIE
Goeral et al. [21]	2017	Prospective study	Neonates with HIE	32	MRI in the first two weeks	cSPO2 and FTOE had no predictive value in predicting outcomes in HIE
Peng et al. [22]	2015	Prospective study	Asphyxiated newborns treated with hypothermia	18	MRI on days 2–3	cSPO2 significantly predicts MRI injury
Lemmers et al. [23]	2013	Prospective study	Neonates with HIE	39	MRI at days 4–6; Griffiths at 1.5 years	cSpo2 significantly predicts the adverse outcomes in 24–72 h
Gucuyener et al. [24]	2012	Prospective study	Asphyxiated newborns treated with hypothermia	10	MRI at first week; Beyleys at two years	cSPO2 and FTOE had no predictive value in predicting outcomes in HIE

Regarding the NIRS characteristics, the studies positioned the NIRS sensors bifrontal (n =2), frontoparietal (n =3), parietal (n =2), and forehead (n =2). The most commonly assessed NIRS measures were regional cSPO2 and FTOE (Table 2).

**Table 2:** HIE and NIRS Characteristics of the Included Studies (n =7) HIE: hypoxic-ischemic encephalopathy, NIRS: near-infrared spectroscopy, cSPO2: cerebral saturation of peripheral oxygen, CBV: cerebral blood volume, TOI: tissue oxygenation index, HbO2: oxyhemoglobin, FTOE: fractional tissue oxygen extraction.

Author	Year	n	Grade of HIE			Location of Sensor	NIRS Device	NIRS Measure Reported
			Mild	Moderate	Severe			
Nakamura et al. [16]	2015	11	4	6	1	Forehead	TRS 10	cSPO2, CBV
Ze et al. [17]	2021	79	0	74	5	Forehead	EGOS-600A	cSPO2
Ancora et al. [18]	2013	12	0	8	4	Bifrontal	NIR0200	TOI
Liu et al. [19]	2020	37	37	0	0	Parietal	NIRScout 1624	HbO2
Niezen et al. [20]	2018	39	0	0	39	Left or right, frontoparietal	INVOS 5100	cSPO2
Goeral et al. [21]	2017	32	0	32	0	Frontoparietal	INVOS5100C	cSPO2, FTOE
Peng et al. [22]	2015	18	0	8	10	Bifrontal	Foresight	cSPO2
Lemmers et al. [23]	2013	39	0	0	39	Frontoparietal	INVOS4100-5100	cSPO2, FTOE
Gucuyener et al. [24]	2012	10	0	6	4	Parietal	NIR0200	TOl, FTOE

Regarding the risk of bias in the included studies, all studies had unclear selection and allocation processes of the patients, index test interpretation, blinding, and outcomes interpretation. On the contrary, low concerns were found regarding the applicability (Table 3).

**Table 3:** Quality Assessment of the Included Studies Using the QUADAS Tool QUADAS: Quality Assessment of Diagnostic Accuracy Studies.

Study ID	Risk of Bias				Applicability Concerns		
	Patient Selection	Index Test	Reference Standard	Flow and Timing	Patient Selection	Index Test	Reference Standard
Nakamura et al. [16]	High	Unclear	High	Low	Low	Low	Low
Ze et al. [17]	Low	Unclear	Low	Low	Low	Low	Low
Ancora et al. [18]	Low	Unclear	Low	Low	Low	Low	Low
Liu et al. [19]	Unclear	Unclear	Unclear	High	Low	Low	Low
Niezen et al. [20]	Low	Unclear	Unclear	Low	Low	Low	Low
Goeral et al. [21]	Low	Unclear	Low	Low	Low	Low	Low
Peng et al. [22]	Unclear	Unclear	Low	Low	Unclear	Low	Low
Lemmers et al. [23]	Low	Unclear	Low	Low	Low	Low	Low
Gucuyener et al. [24]	Unclear	Unclear	Unclear	High	Low	Low	Low

Outcomes

**Figure 2:**
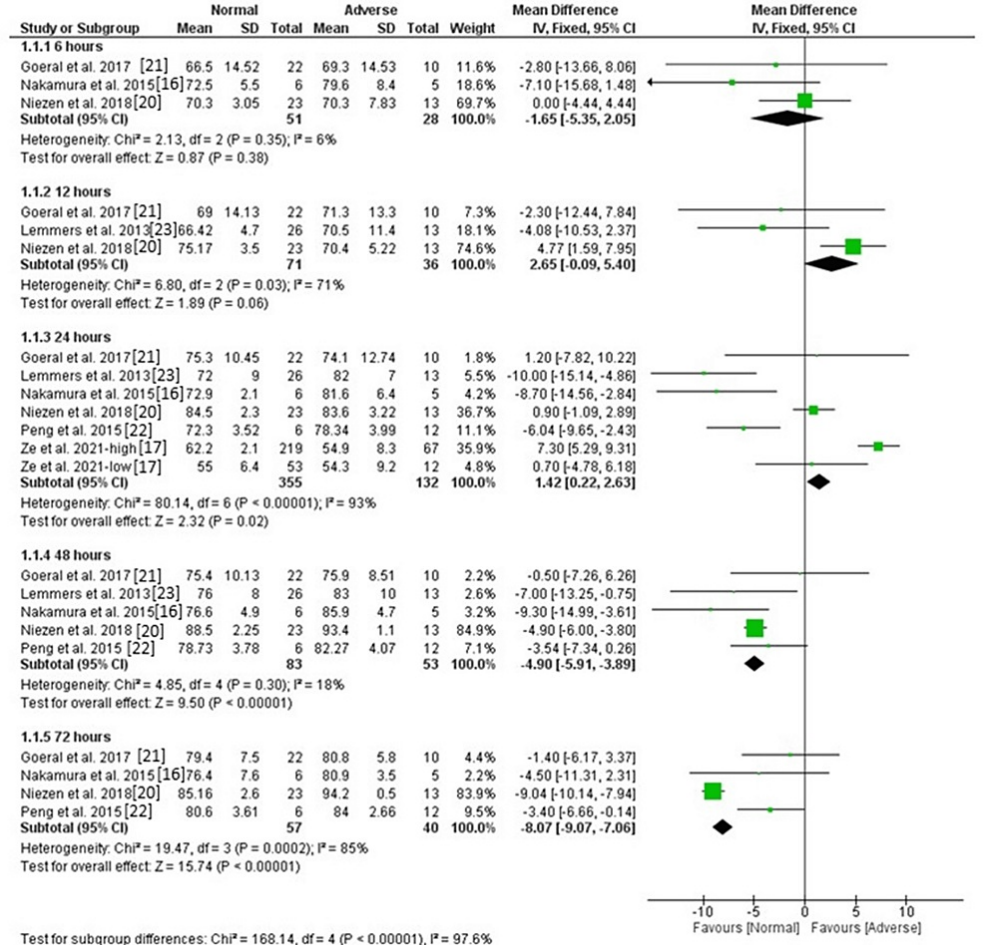
Forest plot of fixed effects of regional cSpO2. cSpO2: cerebral saturation of peripheral oxygen.

**Figure 3:**
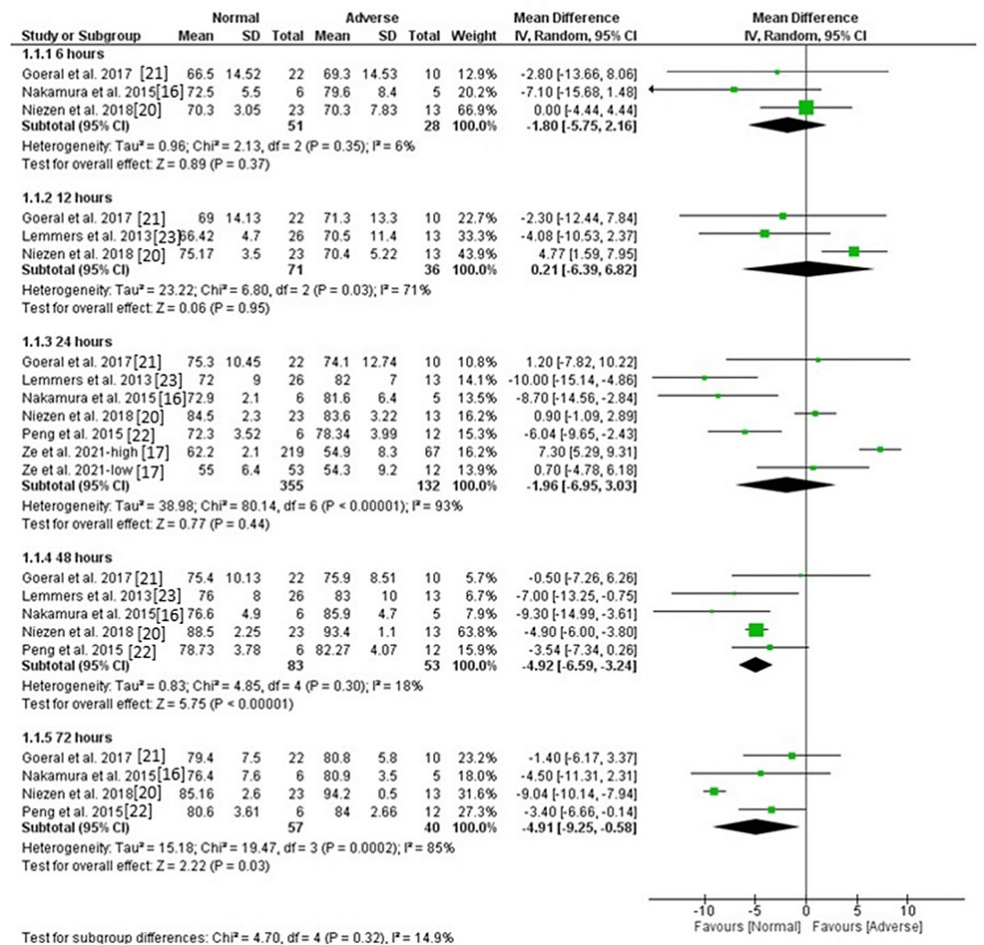
Forest plot of random effects of regional cSpO2. cSpO2: cerebral saturation of peripheral oxygen.

Regional cSpO2: The pooled analysis of three studies showed no significant difference between the normal and abnormal groups in the level of regional cSpO2 [MD = -1.65, 95%CI (-5.35, 2.05), P = 0.38], and the data were homogeneous (P = 0.35, I^2^ = 6%). Similarly, the pooled analysis of 3 and 6 studies showed no significant difference between the two groups after 12 hours and 24 hours groups in the level of regional cSpO2 [MD = 0.21, 95%CI (-6.39, 6.82), P = 0.95] and [MD = -1.96, 95%CI (-6.95, 3.03), P = 0.44], respectively. The data were heterogeneous (P = 0.03, I^2^ = 71%) and (P < 0.00001, I^2^ = 93%). The heterogeneity after 12 hours was resolved by excluding the results of Niezen et al. (P = 0.77, I^2^ = 0), and the difference remained insignificant [MD = -3.57, 95%CI (-9.01, 1.88), P = 0.20], and the heterogeneity after 24 hours could not be resolved. The level of regional cSpO2 after 48 hours was lower in the normal group compared with the abnormal group based on the pooled analysis of five studies [MD = -4.9, 95%CI (-5.91, -3.89), P < 0.00001], and the data were homogeneous (P = 0.30, I^2^ = 18%). Also, the level of regional cSpO2 after 72 hours was lower in the normal group compared with the abnormal group based on the pooled analysis of four studies [MD = -4.91, 95%CI (-9.25, -0.58), P = 0.03], but the data were heterogeneous (P = 0.0002, I^2^ = 85%). The heterogeneity was resolved by excluding the results of Niezen et al. (P = 0.71, I^2^ = 0), and the difference remained significant [MD = -3, 95%CI (-5.5, -0.5), P = 0.02]. Figure 2 and Figure 3 show fixed and random effect models, respectively.

**Figure 4:**
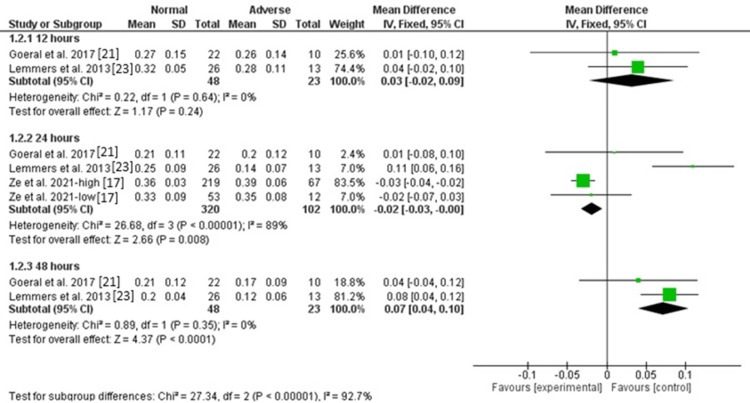
Forest plot of fixed effects of cerebral FTOE. FTOE: fractional tissue oxygen extraction.

**Figure 5:**
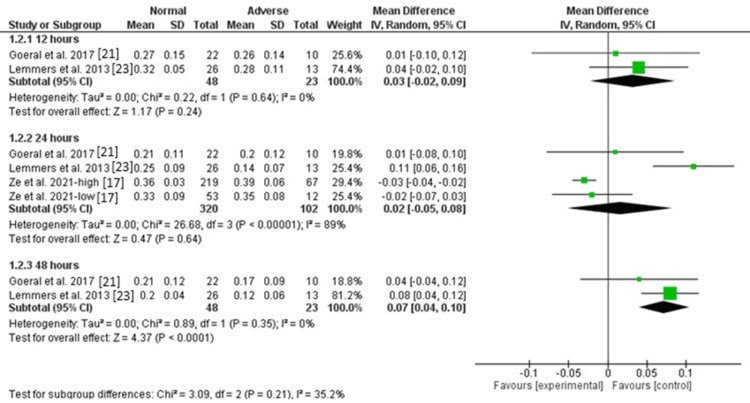
Forest plot of random effects of cerebral FTOE. FTOE: fractional tissue oxygen extraction.

Cerebral FTOE: There was no significant difference between normal and abnormal groups in cerebral FTOE after 12 hours [MD = 0.03, 95%CI (-0.02, 0.09), P = 0.24], and the data were homogeneous (P = 0.64, I^2^ = 0). The pooled analysis of three studies showed no significant difference between the two groups in cerebral FTOE [MD = 0.02, 95%CI (-0.05, 0.08), P = 0.64], but the data were heterogeneous (P < 0.00001, I^2^ = 89%). The heterogeneity was resolved by excluding the results of Lemmers et al. (P = 0.64, I^2^ = 0), and the results showed a significantly lower cerebral FTOE in the normal group compared with the abnormal group [MD = -0.03, 95%CI (-0.04, -0.01), P < 0.001]. On the other hand, the level of cerebral FTOE was higher in the normal group compared with the abnormal group after 48 hours [MD = 0.07, 95%CI (0.04, 0.10), P < 0.0001], and the data were homogeneous (P = 0.35, I^2^ = 0). Figure 4 and Figure 5 show fixed and random effect models, respectively.

Discussion

HIE can lead to several adverse neonatal and developmental outcomes. Thus, accurate outcome prediction and response to treatment are crucial to optimize the management of HIE. Recent reports suggest NIRS monitoring can significantly predict long-term neurodevelopmental outcomes [11]. However, the current evidence regarding the utility of early NIRS monitoring in predicting HIE outcomes is inconclusive. Thus, we conducted the present systematic review and meta-analysis to assess the usefulness of NIRS in predicting the perinatal outcomes of neonates with HIE.

Our analysis showed no significant differences in regional cSpO2 between the normal and abnormal groups at baseline, 12 hours, and 24 hours. However, regional cSpO2 was significantly lower in the normal group compared to the abnormal group at 48 hours and 72 hours. For cerebral FTOE, there was no significant difference between groups at 12 hours. The pooled analysis at 24 hours showed no significant difference, but after removing heterogeneity, cerebral FTOE was significantly lower in the normal group. At 48 hours, cerebral FTOE was significantly higher in the normal group compared to the abnormal group.

Cerebral oxygenation and FTOE were among the primary reported outcomes. FTOE is one of the parameters proposed to predict HIE outcomes [25]. It uses brain oxygenation as a reference due to the cerebral auto-regulation mechanism, which maintains cerebral blood flow even in conditions where ischemia occurs [26]. FTOE represents the balance between oxygen delivery to the gut and gut oxygen consumption [27]. This formula is calculated using (SpO2-rSO2)/SpO2, where SpO2 stands for arterial oxygen saturation, and rSO2 stands for splanchnic tissue oxygenation [28].

On the other hand, TOI represents cerebral venous saturation and might be used to monitor cerebral oxygenation. TOI and FOE were shown to be highly linked [29].

This systematic review and meta-analysis utilized observational studies to quantitatively reflect the predictive value of NIRS monitoring for HIE outcomes. However, the present study has certain limitations. Our review was limited to human studies only and excluded pre-clinical evidence. Furthermore, the search was limited to studies published in the English language, possibly introducing publication bias. The sample size of the included studies in the present meta-analysis was relatively small. The small number of included studies did not allow us to compare different commercially available devices used in these studies and the placement of sensors. We could also not control for the potential confounders that may interfere with NIRS monitoring, such as hair, type of spotlight, hematoma, and movement artifacts.

## Conclusions

In conclusion, early cerebral NIRS monitoring is beneficial in predicting immediate outcomes of HIE in term neonates. Our analysis showed that several NIRS parameters, such as regional cSpO2 and cerebral FTOE, are significantly associated with adverse outcomes in the first 72 hours of birth. However, the current literature is still limited by the low number of prospective studies and low quality of evidence. Thus, larger prospective studies are still needed to confirm the clinical utility of early NIRS monitoring for predicting HIE outcomes.
